# Birds and Influenza H5N1 Virus Movement to and within North America

**DOI:** 10.3201/eid1210.051577

**Published:** 2006-10

**Authors:** John H. Rappole, Zdenek Hubálek

**Affiliations:** * Smithsonian Institution, Washington, DC, USA;; † Academy of Sciences, Valtice, Czech Republic

**Keywords:** Avian Influenza A, Orthomyxovirus, Migration, Birds, HPAI, H5N1, Movement, Western Hemisphere, Perspective

## Abstract

Highly pathogenic avian influenza (HPAI) H5N1 expanded considerably during 2005 and early 2006 in both avian host species and geographic distribution. Domestic waterfowl and migratory birds are reservoirs, but lethality of this subtype appeared to initially limit migrant effectiveness as introductory hosts. This situation may have changed, as HPAI H5N1 has recently expanded across Eurasia and into Europe and Africa. Birds could introduce HPAI H5N1 to the Western Hemisphere through migration, vagrancy, and importation by people. Vagrants and migratory birds are not likely interhemispheric introductory hosts; import of infected domestic or pet birds is more probable. If reassortment or mutation were to produce a virus adapted for rapid transmission among humans, birds would be unlikely introductory hosts because of differences in viral transmission mechanisms among major host groups (i.e., gastrointestinal for birds, respiratory for humans). Another possible result of reassortment would be a less lethal form of avian influenza, more readily spread by birds.

## Abstract 2

TOC Summary: Migratory birds are unlikely introductory hosts for this highly pathogenic virus in its present form into North America.

Avian influenza virus A refers collectively to a group of viruses within the family *Orthomyxoviridae* that has a worldwide distribution and causes a variety of diseases in birds. Classification of influenza viruses is based on 2 glycoproteins (antigens) characteristic of the group members: hemagglutinin, of which 16 forms are known; and neuraminidase, of which 9 forms have been described. In 1997, a virulent, highly pathogenic avian influenza (HPAI) A virus, identified as the H5N1 subtype, was identified in samples taken in Hong Kong (*1**,**2*). This virus has spread to several localities in Asia and, since late 2005, Europe (*3*) and Africa (*4*) (Table 1). HPAI H5N1 virus is found most commonly in domestic fowl, although as of late 2005, it has been found in migratory and resident birds of several orders (mainly Anseriformes) and in pigs, civets, house cats, tigers, leopards, and humans (*3*). This virus poses a potential danger to human populations; 224 human cases of H5N1 avian influenza have been reported as of May 29, 2006; 127 of these cases were fatal (*17*). Its discovery in migratory birds is especially troubling because of the potential for rapid dispersal of the virus across continents and hemispheres.

**Table 1:** Geographic spread of highly pathogenic avian influenza H5N1 subtype since 1996

Date	Event
1996	1st isolation; domestic geese, southern China (*5*)
1997–1998	Chickens, Hong Kong; 18 humans (6 deaths) (*6*)
1999	Geese, Hong Kong (*7*)
2001	Geese from China in Vietnam (*8*)
Nov 2002	Hong Kong poultry, other bird species in or near zoologic parks (*7*)
Feb 2003	Human travelers from Fujian Province (China) (*9*)
Dec 2003–Nov 2005	Poultry (mainly chickens) and humans: South Korea, Vietnam, Thailand, Hong Kong, Cambodia, Laos, Indonesia, China, and Malaysia (*6*)
Jan 2004	Wild birds: Hong Kong (*10*)
Feb 2004	Birds in a zoo collection: Cambodia (*10*)
Mar 2004	Wild bird: South Korea (*10*)
Oct 2004	Bird smuggled from Thailand into Belgium (*11*)
Apr–Jun 2005	Migratory birds: Qinghai Lake and Xinjiang Province, China (*12*)
Jul–Oct 2005	Poultry and wild waterfowl: Novosibirsk, Altai, Kurgansk, Omsk, and Tyumen regions, Asian Russia (*13**,**14*)
Aug 2005	Geese and other poultry: northern Kazakhstan, Tibet (*13*)
Aug 2005	Migratory waterfowl: northern Mongolia (*15*)
Aug–Oct 2005	Poultry and pigeons: Ural Territory, Russia (*13*)
Aug 2005	Wild waterfowl: Kalmykia, European Russia (*13*)
Oct 2005	Domestic turkeys: Western Asian turkey (*13*)
Oct–Nov 2005	Poultry and wild migratory birds: Romania, Ukraine (*13*)
Oct 2005	Wild birds: Thailand (*15*)
Oct–Nov 2005	Poultry, wild birds, some humans: 7 Chinese provinces (*15*)
Oct 2005	Migratory waterfowl: Croatia (*13*)
Oct 2005	Poultry: Tula and Tambov regions, European Russia (*14*)
Oct 2005	Quarantined birds from Taiwan in United Kingdom (*16*)
Jan 2006	Humans: Iraq (*15*)
Jan 2006	Poultry: Nigeria, India (Maharashtra) (*15*)
Feb 2006	Migratory waterfowl: Bulgaria, Greece, Italy, Slovenia, Bosnia, Azerbaijan, Iran, Georgia, Germany, Switzerland, Austria, Hungary, France, Croatia, Slovakia, Bosnia (*15*)
Feb 2006	Poultry: Egypt, Cameroon, Niger, Ethiopia (*15*)
Mar 2006	Migratory birds: Sweden, Denmark, Serbia, Poland, Czech Republic (*15*)
Mar 2006	Poultry: Afghanistan, Pakistan, Albania, Israel, Jordan, Lebanon (*15*)
Apr 2006	Poultry: Burkina Faso, Côte d'Ivoire, Myanmar, Nigeria, Palestinian Autonomous Territories (*15*)
May 2006	Poultry: Sudan; migratory birds: United Kingdom (*15*)

We review facts concerning outbreaks of H5N1; the species of birds, especially migrants, known to have been infected by this subtype; and available information on the ability of migrants to serve as reservoir or introductory hosts that move the virus from outbreak areas to new localities. On the basis of this information, we consider the avian pathways by which HPAI H5N1 might enter the Western Hemisphere and, once present, the likelihood that it will be able to disperse to new regions. We define migratory or migrant birds as those species that move annually between geographically separate breeding and wintering quarters. Migrating birds are those actually in the process of moving from 1 locality to another.

## Ecology of Influenza A Viruses

**Figure 1:**
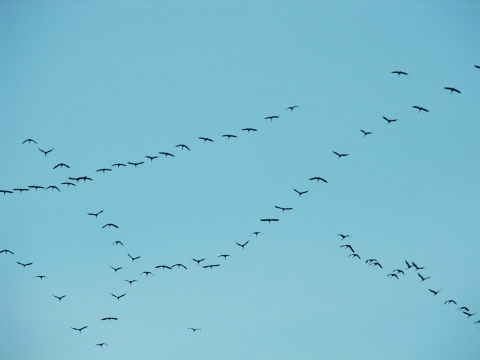
Saurus cranes (*Grus antigone*) over Naung Mung, Myanmar, in March 2006.

Avian influenza A viruses are common and widespread in birds. Most viruses in this family attack the intestinal tract of the host preferentially and are spread mainly by shedding in host feces (*18**,**19*). Waterfowl, e.g., ducks, geese, and swans (Anseriformes), and shorebirds (Charadriiformes) are particularly susceptible because they are exposed to water that may be contaminated with infected fecal matter, especially at specific sites and seasons, when these birds congregate densely at relatively confined and shallow water bodies (Figure 1). A secondary mode of viral spread is consumption of infected avian host parts by predators, including captive carnivores, avian raptors, and carrion-feeding vertebrates. Infection by most avian influenza A strains appears to be asymptomatic for the host (*18*). Proportions of birds shedding active virus can be high (e.g., >30% in some Canadian duck populations) among juvenile waterfowl gathered in large flocks on lakes and ponds during the summer postbreeding molting period but decrease rapidly during southward migration, falling to 1% to 2% during winter (*18*). Nevertheless, shedding of active virus can remain as high as 0.25% by individual birds among northbound spring migrants, sufficient to reinfect northern breeding populations (*18*).

Most birds appear to be more or less susceptible to >1 strain of avian influenza A, but rates of infection and levels of susceptibility to the different viral subtypes vary among taxa. For instance, H3 and H6 subtypes are common in ducks, geese, and swans (Anseriformes), while H4, H9, H11, and H13 subtypes are more prevalent in sandpipers, terns, and gulls (Charadriiformes) (*20*). The best opportunities for viral transmission among large numbers of anseriform hosts would likely be on lakes and ponds in summer, where large concentrations gather for weeks to undergo the postbreeding, premigratory molt (*18*). For charadriiformes, the greatest viral transmission opportunities would likely be at stopover sites during fall migration, where tens of thousands of individual birds congregate to feed and roost (*20*).

## Avian Influenza in Humans

Humans and other mammals normally are not susceptible to infection by avian influenza A viruses. Nevertheless, several subtypes of avian influenza or bird-origin influenza viruses have infected humans; 3 of these subtypes have caused pandemics within the past century. At present, HPAI H5N1 is entirely an avian influenza subtype. Humans can become infected, but so far as is known, they must inhale or ingest massive viral doses from excreta or tissues of infected birds to do so. Although clinically ill humans have high death rates, ≈50%, passage of H5N1 virus from human to human is rare (*3*).

The more humans infected with HPAI H5N1, the greater the probability that reassortment with a human influenza virus strain will occur and produce a lethal form that is spread readily between humans (*18**,**19*). However, viral interhost transmission strategies differ fundamentally for those viruses that primarily infect humans versus those that infect birds. Bird viruses have an affinity for the host's intestinal tract, and interhost transmission occurs mainly by fecal contamination of shared water bodies. Human viruses more often attack the respiratory system and depend on shedding in respiratory effluvia for interhost transfer. If, or when, a reassortment or mutation of HPAI H5N1 produces a virus capable of efficient horizontal transfer among humans, the new virus would likely not be particularly effective in transfer among birds; migrants likely would play little role in spread of such a virus. Vaccines produced to prevent human infection by H5N1 might not be effective against a new virus produced by reassortment.

## Birds as HPAI H5N1 Reservoirs and Introductory Hosts in the Old World

The main reservoirs and introductory hosts for avian influenza A viruses in general are migratory waterfowl and domestic fowl (*18**,**19*). HPAI H5N1, however, causes high rates of disabling illness and death in most avian species (*21*). High rates of illness would prevent migrants from being introductory hosts, since sick wild birds normally cannot move far and do not survive long. Thus, perhaps not surprisingly, no evidence exists that migrants were introductory hosts for H5N1 for several years after its initial appearance in Guangdong Province, People's Republic of China, in 1996. In fact, no deaths or even infections of migrants were reported until December 2002, when several migrants and exotic birds were found dead at a Hong Kong park and zoologic garden (*10*). Of 3,095 outbreaks of HPAI H5N1 reported from December 2003 through February 2005, all involved captive birds or domestic fowl (*6*). Until early August 2005, only 2 outbreaks of HPAI H5N1 had been confirmed in migratory birds presumed to be completely separate from domestic fowl: Qinghai Lake and Xinjiang Province, China, (April, May 2005) (*12*) and Lakes Erhel and Khunt in northern Mongolia (August 2005) (*15*). However, that situation has changed, and several new outbreaks have been recorded in migrants that were presumably separate from domestic fowl within the last few months (Appendix Table), perhaps signaling genetic modification of the virus (*19*).

Data based on observations of dead wild birds at sites where infections have broken out and negative results from subsequent extensive screening for seropositive or infected migrants around outbreak sites have indicated that HPAI H5N1 was lethal for most wild birds, at least until recently. Nevertheless, some studies have demonstrated that chickens, domestic ducks, and geese infected under laboratory conditions, as well as some wild birds exposed under quasilaboratory conditions (e.g., birds fed, watered, and protected at zoologic parks or gardens), survive infection and shed the virus in active form (*10**,**22**,**23*). The work by Komar et al. (*24*) on wild birds exposed to West Nile virus (WNV) under laboratory conditions may be instructive in this regard. These researchers found that in species like the fish crow (*Corvus ossifragus*), in which individual birds were known to have high death rates on exposure to the New York 99 subtype of WNV in the wild (on the basis of large numbers of birds found dead and failure to find free-flying birds captured that were seropositive), survival rates from exposure in the laboratory were 45%. When one considers that birds kept in a laboratory have ready access to food and water during their illness, as well as protection from inclement weather and predators, this finding perhaps is not surprising. However, wild birds associating with free-ranging domestic fowl at farm ponds, or captive exotic birds at city parks or zoological gardens, may receive some of the same benefits as laboratory birds, experiencing conditions conducive to survival of infection by HPAI H5N1.

Recent detections of HPAI H5N1 in free-ranging migrants may be a result of heightened awareness and thus the virus could have been circulating in migrants, although undetected. This explanation is unlikely considering the extensive screening of blood and feces of migrants in the past several years in Europe, parts of Asia, and North America. These screenings have searched for birds seropositive for H5N1 and other avian influenza type A viruses. These searches have involved sampling thousands of birds of hundreds of species (*25**,**26*). The virus may also have changed to some degree (*2**,**19*), allowing higher survival rates among some species of migrants. Both explanations may have some relevance to the current situation. In any event, some migratory birds may now be able to move HPAI H5N1 in active form over considerable distances (Table A1). Increasing numbers of recent reports document apparent movement of the virus, whereas before April 2005, no evidence existed of HPAI H5N1 in free-ranging migratory birds distant from domestic fowl, despite years of sampling of tens of thousands of migratory waterfowl of several species from wetland sites across the European continent (*25*).

## Possible Role of Birds in Arrival of HPAI H5N1 Avian Influenza in New World

To date, HPAI H5N1 has not been recorded in the New World, although outbreaks of related avian influenza viruses lethal to domestic fowl have occurred in Ontario, Canada, in 1966 (H5N9); Pennsylvania, United States in 1983 (H5N2); Puebla, Mexico, in 1994 (H5N2); Chile in 2002 (H7N3); Canada in 2004 (H7N3); and Texas, United States, in 2004 (H5N2) (*27*). All of these outbreaks occurred in domestic poultry and were controlled without further diffusion. We see 3 possible modes by which HPAI H5N1 might gain entry to the New World if birds were the introductory host: 1) normal interhemispheric migration, 2) vagrancy, and 3) legal and illegal importation of birds as explained in the following section.

### Normal Interhemispheric Migration

**Figure 2:**
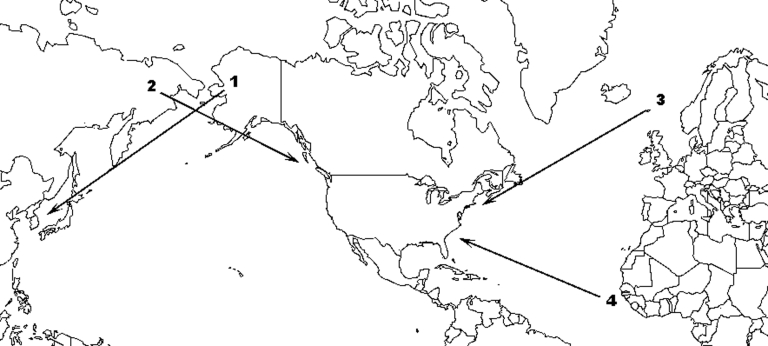
Map of known routes for natural interhemispheric bird movement: route 1, migrants breeding in Alaska and wintering in East Asia; route 2, migrants breeding in East Asia and wintering along the Pacific Coast of North America; route 3, migrants breeding in Iceland or northwestern Europe and wintering along the Atlantic Coast of North America; route 4, vagrants from West Africa carried by tropical storm systems across the Atlantic to eastern North America.

Few individual birds within few species undertake regular, interhemispheric migration. However, some do, and the waterfowl (Anseriformes, Charadriiformes, Ciconiiformes) could be introductory hosts for HPAI H5N1 to the New World (Table 2). Three pathways are used annually by a small number of waterfowl species to travel between the hemispheres: 1) Alaska–East Asia, in which birds that breed in Alaska winter in East Asia; 2) East Asia–Pacific North America, in which birds that breed in northeast Asia winter along the Pacific Coast of North America; and 3) Europe–Atlantic North America, in which birds that breed in Iceland or northwestern Europe winter along the Atlantic Coast of North America (Figure 2, Table 2).

**Table 2:** Known interhemispheric movement by migratory or vagrant waterfowl (Ciconiiformes, Anseriformes, Charadriiformes), domestic bird trade (Galliformes), or exotic bird trade (Galliformes, Psittaciformes) from Eurasia to North America*

Species	Likely mode of entry
Bean goose (*Anser fabalis*)	Migration†
**Greylag goose** (*A. anser*) (domestic)	Exotic and domestic bird trade
**Whooper swan** (*Cygnus cygnus*)	Migration†
Falcated duck (*Anas falcata*)	Migration,† exotic bird trade, zoos, vagrant
**Eurasian wigeon** (*A. penelope*)	Migration,†‡ exotic bird trade, zoos
**Mallard** (*A. platyrhynchos*) (domestic and wild)	Exotic and domestic bird trade
Garganey (*A. querquedula*)	Migration,†‡ exotic bird trade, zoos
Green-winged teal (*A. crecca*)	Migration†‡
**Common pochard** (*Aythya ferina*)	Migration†
**Tufted duck** (*Aythya fuligula*)	Migration†‡
**Smew** (*Mergellus albellus*)	Migration†
**Jungle fowl** (*Gallus gallus*) (domestic)	Domestic bird trade
**Pheasants** (Phasianidae)	Exotic bird trade, zoos
**Quail** (*Coturnix coturnix*)	Domestic bird trade
**Wild turkey** (*Meleagris gallopavo*) (domestic)	Domestic bird trade
Red-faced cormorant (*Phalacrocorax urile*)	Migration§
**Gray heron** (*Ardea cinerea*)	Vagrant
**Little egret** (*Egretta garzetta*)	Vagrant
Cattle egret (*Bubulcus ibis*)	Vagrant
Eurasian kestrel (*Falco tinnunculus*)	Vagrant
Northern lapwing (*Vanellus vanellus*)	Vagrant
Mongolian plover (*Charadrius mongolus*)	Migration†
Common ringed plover (*C. hiaticula*)	Migration§
Eurasian dotterel (*C. morinellus*)	Migration§
Spotted redshank (*Tringa erythropus*)	Migration†
Wood sandpiper (*T. glareola*)	Migration†
Gray-tailed tattler (*Heteroscelus brevipes*)	Migration†
Bar-tailed godwit (*Limosa lapponica*)	Migration§
Red-necked stint (*Calidris ruficollis*)	Migration§
Little stint (*C. minuta*)	Vagrant
Sharp-tailed sandpiper (*C. acuminata*)	Migration†§
Ruff (*Philomachus pugnax*)	Migration†‡
Little gull (*Larus minutus*)	Migration‡
**Black-headed gull** (*L. ridibundus*)	Migration†‡
Black-tailed gull (*L. crassirostris*)	Vagrant
Yellow-legged gull (*L. cachinnans*)	Vagrant
Slaty-backed gull (*L. schistisagus*)	Migration†
Common tern (*Sterna hirundo*)	Vagrant
**Rock pigeon** (*Columba livia*) (domestic)	Exotic bird trade
Oriental turtle-dove (*Streptopelia orientalis*)	Exotic bird trade
European turtle-dove (*S. turtur*)	Exotic bird trade
Eurasian collared-dove (*S. decaocto*)	Exotic bird trade
**Parrots** (*Psittacidae*)	Exotic bird trade

*Species shown in **boldface** are known to have been infected with highly pathogenic avian influenza H5N1. Sources for information on migrant or vagrant status are Kessel and Gibson (*28*), Palmer (*29*), and the American Ornithologists' Union (*30*). Nomenclature follows the American Ornithologists Union checklist (*30*) to the degree possible. Supplementary source: Rasmussen and Anderton (*31*). †Route 2. See Figure 2. ‡Route 3. See Figure 2. §Route 1. See Figure 2.

Two lines of evidence argue against normal, interhemispheric migration as a likely mode of entry for HPAI H5N1 into the Western Hemisphere. First, as discussed previously, data indicate that most infected individual birds of most species of migrants become extremely ill and either cannot migrate far in their weakened state or die at the place of infection. Second, investigation of the genetics of avian influenza viruses has shown that little natural interchange occurs between the Eastern and Western Hemispheres: each hemisphere appears to have an avian influenza virus community that is largely distinct (*18*). This fact is particularly noteworthy when one considers that most avian influenza A viruses appear to be asymptomatic, and migrants readily transport them in infectious form, in stark contrast to the situation for HPAI H5N1. Presumably, the distinct nature of the avian influenza A community in each hemisphere results from the fact that the main reservoir for these viruses is migrants, and few migrants move regularly between the hemispheres (*32*).

### Vagrancy

Perhaps a third or more of Eurasian waterfowl species have traveled into the Western Hemisphere as vagrants; some occur more regularly than others, including those listed in Table 2. However, all Eurasian vagrants are, by definition, extremely rare in the New World (a few birds per decade). One mode of interhemispheric vagrancy is tropical storm systems that originate off the West African coast during the Atlantic hurricane season, which lasts from June to November each year. These systems can, and occasionally do, sweep up and transport Old World birds, especially waterfowl, across the Atlantic to the New World (route 4, Figure 2). Vagrancy is much rarer (by several orders of magnitude) than normal interhemispheric migration and seems an even less likely mode of entry for HPAI H5N1.

### Legal and Illegal Importations

Human traffic in birds and bird products is the sole documented means of HPAI H5N1 movement between geographically separate regions to date (*19*). While migratory birds have been suspected of involvement, particularly in cases in which no obvious human interchange of infected birds or products has occurred, these conclusions are inferred (*19*). Thus, if HPAI H5N1 is to be kept out of the Western Hemisphere, control of legal and illegal imports should be the primary focus of prevention efforts.

The legal importation of exotic birds has declined dramatically in the United States since enactment of the 1992 Wild Bird Conservation Act. Nevertheless, 2,770 birds entered the country through the New York port of entry in 1999, including 323 pet birds and 2,447 commercial birds. In addition, 12,931 birds passed through in transit (S. Kaman, US Department of Agriculture [USDA], pers. comm.) Legal importations are controlled by USDA Animal and Plant Health Inspection Service and the US Fish and Wildlife Service. Most imported birds undergo a 30-day quarantine at USDA facilities located near each of the 3 allowed ports of entry: New York, Miami, and Los Angeles. Quarantine procedures include isolation in indoor, air-filtered cages and standard testing for common poultry diseases, including avian influenza. The number of illegally imported birds is not known. These birds are not subject to quarantine and testing and could be a mode of entry for HPAI H5N1. Hawk eagles from Thailand infected with the virus were recently detected while being smuggled into Belgium (11). Although these birds were detected and quarantined, they serve as an example of how such imports could spread the virus. Species commonly associated with the transhemispheric bird trade are listed in Table 2.

If birds turn out to be responsible for entry of HPAI H5N1 into the Western Hemisphere, illegal import of an infected bird or bird product seems the most likely mode of entry. We base this conclusion on the fact that illegally imported birds, unlike infected, free-flying migrants, are provided food and water ad libitum and protected from predators, greatly increasing their chances of survival in an infectious state. Furthermore, these birds often end up in close association with other, similarly protected birds, sharing the same food or water, a situation that provides ample opportunity for viral transmission.

## Possible Role of Birds in Movement of HPAI H5N1 in Western Hemisphere

Movement of HPAI H5N1 by sale of infected domestic fowl or poultry products in the United States and Canada is unlikely, given existing regulations. Thus, a major mode of HPAI spread available in much of Eurasia would be ruled out. Also, most domestic fowl are kept separate from wild migratory waterfowl in both countries, which would rule out a second major mode of introduction and cross-infection. Mixing of wild migratory birds with captive, exotic birds is relatively common, however, at North American zoos. Birds in such exhibits should be screened regularly for H5N1 or whatever HPAI virus is in circulation during a given year.

The HPAI H5N1 subtype of avian influenza A causes high mortality rates in most wild birds, at least in its present form. The situation is similar to that found for the form of WNV introduced into the Western Hemisphere in 1999 (*24**,**32**–**34*). Even under conditions in which food, water, and protection from predators are provided, death rates are high. These kinds of death rates could result if the current form of HPAI H5N1 were introduced into New World bird populations. In such a scenario, migrants might not be capable of moving the virus far from its point of introduction, at least initially. Also, the die-offs occurring at the site of entry likely would be obvious to wildlife disease monitors, which would allow for rapid quarantine. However, if the H5N1 virus were introduced into the Western Hemisphere, migratory birds, particularly anseriforms (ducks, swans, geese), might serve as dispersal agents, especially if the virus were to change to a less lethal form through reassortment or mutation.

A key difference between mosquitoborne WNV and birdborne HPAI H5N1 is the virtual absence of effective reservoir hosts other than birds for the latter. WNV can be maintained without birds because infected mosquitoes can pass active virus to subsequent generations through vertical transmission (*35*). So far as is known, no alternative to birds exists as major reservoir hosts for HPAI H5N1.

An additional consideration concerning the future of HPAI H5N1, should it gain wide circulation in migratory birds, is the possibility of infection of a bird already infected with another form of avian influenza virus. Such infection could result in reassortment and production of a new virus, possibly less lethal than HPAI H5N1 but more readily spread.

## Conclusions

HPAI H5N1 spread rapidly across Eurasia during 2005 for reasons that are not entirely understood. Despite this rapid movement, effective introduction (i.e., under conditions allowing its spread) of the virus to the New World through migratory or vagrant birds seems unlikely. Few individual members of few waterfowl species migrate between hemispheres, and should a bird make the journey while shedding sufficient active virus to infect birds in the Western Hemisphere, newly infected birds would probably die before being able to transport the virus from the entry site. If spread of HPAI H5N1 to the New World occurs in its current form (e.g., through domestic or pet bird trade or smuggling), it should be readily detectable because of the large number of dead native birds likely to result. However, the virus is changing (*19*), and a modified H5N1 virus introduced into the Western Hemisphere could be moved more readily by migratory waterfowl. If this event were to occur, the virus should be amenable to control through isolation and quarantine. If viral reassortment or mutation occurs to produce a new virus that is readily transmissible to humans, the role of birds in general and migrants in particular may be moot because of the fundamentally different methods of infection favored by viruses infecting humans and birds. Viruses infecting birds preferentially attack the intestinal tract and are shed with the feces; by contrast, human viruses mainly attack the respiratory tract and are shed with respiratory effluvia. If HPAI H5N1 were to gain wide circulation among migrants, it might infect a bird already infected with another form of avian influenza A and undergo reassortment to produce a low-pathogenic form that is more readily spread.

## Appendix

**Table A1:** Wild bird species confirmed to have been infected with highly pathogenic avian influenza H5N1*

Species	Localities and dates
"Wild birds"†	Siberia, Russia, Aug 2005 (*1*)
"Wild birds"†	Kazakhstan, Aug 2005 (*1*)
**Great crested grebe** (*Podiceps cristatus*)‡	Siberia, Aug 2005 (*2*)
**Black-necked swan** (*Cygnus melanocoryphus*)§	Hong Kong, Dec 2002 (*3*)
**Whooper swan** (*Cygnus cygnus*)‡	Mongolia, Aug 2005; Germany, Denmark, and Iran, Feb 2005 (*1**,**2*)
**Mute swan** (*Cygnus olor)*‡	Romania, Croatia, Oct 2005; Russia (Volga delta), Nov 2005; Bulgaria, Greece, Italy, Slovenia, France, Germany, Austria, Switzerland, Hungary, Bosnia, Azerbaijan, Georgia and Iran, Feb 2006; Denmark, Serbia, Poland, and Czech Republic, March 2006 (*1**,**2*)
**Coscoroba swan** (*Coscoroba coscoroba*) §	Hong Kong, Dec 2002 (*3*)
**Bar-headed goose** (*Anser indicus)* †‡	Hong Kong, Dec 2002; Qinghai Lake, China, Apr 2005; Mongolia, Aug 2005 (*1**,**3**,**4*)
**Lesser white-fronted goose** (*Anser erythropus*)‡	Romania, Oct 2005 (*2*)
**Greylag goose** (*Anser anser*)‡	Denmark and Germany, March 2006 (*2*)
**Canada goose** (*Branta Canadensis*§¶	Hong Kong,¶ Dec 2002 (*3*)
**Red-breasted goose** (*Branta ruficollis*)‡	Greece (Skyros Island), Feb 2006 (*2*)
**Ruddy shelduck‡**(T*adorna ferruginea*)‡	Mongolia, Aug 2005, Kalmykia, Aug 2005 (*2**,**5*)
**Wood duck** (*Aix sponsa*)¶	Hong Kong, Dec 2002 (*3*)
**Argentine shoveller** (*Anas platalea*)§	Hong Kong, Dec 2002 (*3*)
White-cheeked pintail (*Anas bahamensis*)§	Hong Kong, Dec 2002 (*3*)
Chestnut-breasted teal (*Anas castanea*)#	Hong Kong, Dec 2002 (*3*)
Chiloe wigeon (*Anas sibilatrix*)§	Hong Kong, Dec 2002 (*3*)
**Mallard** (*Anas platyrhynchos*)‡	Siberia, Aug 2005; Germany, Switzerland, and Hungary, Feb 2006 (*2**,**5*)
**"Wild ducks"** (*Anas* sp.)‡	Siberia, Aug 2005, Kalmykia, Aug 2005; Romania, Oct 2005; Crimea, Dec 2005; Germany, Switzerland, Ukraine, and Turkey, Feb 2006; Cameroon, March 2006 (*1**,**2**,**5*)
**Gadwall** (*Anas strepera*)‡	Siberia, Aug 2005 (*5*)
**Eurasian wigeon** (*Anas penelope*)‡	Mongolia, Aug 2005 (*2*)
Maned wood duck (*Cheonetta jubata*)#	Hong Kong, Dec 2002 (*3*)
**Common pochard** (*Aythya ferina*)‡	Siberia, Aug 2005; France, Feb 2006; Switzerland and Germany, March 2006 (*2**,**5*)
**Tufted duck** (*Aythya fuligula*)‡	South Sweden, Denmark, Germany, and Switzerland, March 2006 (*2*)
**Scaup** (*Aythya marila*)‡	South Sweden, March 2006 (*2*)
**Red-crested pochard** (*Netta rufina*)†	Hong Kong, Dec 2002 (*3*)
Rosy-billed pochard (*Netta peposaca*)§	Hong Kong, Dec 2002 (*3*)
**Ruddy duck** (*Oxyura jamaicensis*)¶	Hong Kong, Dec 2002 (*3*)
**Goosander** (*Mergus merganser*)§	Poland, March 2006 (*2*)
**Smew** (*Mergus albellus*)‡	Slovakia, Feb 2006 (*2*)
**Little cormorant** (*Phalacrocorax niger*)†	Thailand, Dec 2004 (*1*)
**Great cormorant** (*Phalacrocorax carbo*)†‡	Qinghai Lake, People's Republic of China, Apr 2005 (*4*)
**Grey heron** (*Ardea cinerea*)†‡	Hong Kong, Dec 2002, Jan 2004, Dec 2004; Romania, Oct 2005 (*1**–**3*); Slovenia, Feb 2006 (*2*)
**Little egret** (*Egretta garzetta*)†‡	Hong Kong, Dec 2002 (*3*); Hong Kong, Jan 2006 (*2*)
**Chinese pond** Heron (*Ardeola bacchus*)†	Hong Kong, Jan 2005 (*1*)
**Asian openbill** (*Anastomus oscitans*)†	Thailand, Dec 2004 (*1*)
**Greater flamingo** (*Phoenicopterus ruber*)†**	Hong Kong, Dec 2002; Kuwait, Nov 2005 (*3**,**5*)
Grey-headed fish-eagle (*Ichthyophaga ichthyaetus*)†	Cambodia, Feb 2004 (*1*)
"Serpent eagle" (*Spilornis sp.*)†	Cambodia, Feb 2004 (*1*)
"Hawk-eagle" (*Spizaetus* sp)†	Cambodia, Feb 2004 (*1*)
Mountain hawk-eagle (*Spizaetus nipalensis*)†	Thailand, Oct 2004 (*6*)
**Common buzzard** (*Buteo buteo*)‡	Denmark and Germany, March 2006 (*2*)
**Rough-legged buzzard** (*Buteo lagopus*)‡	Denmark. March 2006 (*2*)
**Goshawk** (A*ccipiter gentilis*)‡	Germany, Feb 2006 (*2*)
**Peregrine falcon** (*Falco peregrinus*)†‡	Hong Kong, Mar 2003, Jan 2004; Slovakia, Feb 2006; Denmark and Germany, March 2006 (*1**,**2*)
**Saker falcon** (*Falco cherrug*)†	Saudi Arabia, Jan 2006 (*2*)
**Kestrel** (*Falco tinnunculus*)‡	Germany, March 2006 (*2*)
**Coot** (*Fulica atra*)‡	Siberia, Aug 2005; Kalmykia Aug 2005 (*5*); Switzerland and Germany, March 2006 (*2*)
**Common moorhen** (*Gallinula chloropus*)***.*	Romania, Oct 2005 (*2*)
**Great black-headed gull** (*Larus ichthyaetus*)‡	Qinghai Lake, China, Apr 2005 (*4*)
**Brown-headed gull** (*Larus brunnicephalus*)‡	Qinghai Lake, China, Apr 2005 (*4*)
**Black-headed gull** (*Larus ridibundus*)†‡	Hong Kong, Dec 2002 (*3*); Hungary, Feb 2006 (*2*)
"Gulls" (*Larus spp*)‡*.*	Crimea, Dec 2005; Croatia, March 2006 (*2*)
"Pigeons" (Columbiformes)†	Thailand, Dec 2004 (*1*)
Rock pigeon (*Columbia livia f. domestica*)†**	Hong Kong, Dec 2002; Thailand, 2005; Chelyabinsk, Russia, Aug 2005 (*2**,**3**,**5*); Crimea, Dec 2005 (*2*)
Red-collared dove (*Streptopelia tranquebarica*)†	Thailand, Dec 2004 (*1*)
Eagle owl (*Bubo bubo*)‡	Sweden, March 2006 (*2*)
Forest eagle-owl (*Bubo nipalensis*)†	Cambodia, Feb 2004 (*1*)
Brown fish-owl (*Ketupa zeylonensis*)†	Cambodia, Feb 2004 (*1*)
Buffy fish-owl (*Ketupa ketupu*)†	Cambodia, Feb 2004 (*1*)
Spotted wood-owl (*Strix seloputo*)†	Cambodia, Feb 2004 (*1*)
"Parrots" (Psittaciformes)†	Cambodia, Feb 2004 (*1*)
Black drongo (*Dicrurus macrocercus*)†	Thailand, Dec 2004 (*1*)
House crow (*Corvus splendens*)‡	Hong Kong, Jan 2006; Afghanistan and Pakistan, March 2006 (*2*)
Large-billed crow (*Corvus macrorhynchus*)‡	Hong Kong, Jan 2006 (*2*)
**Carrion crow** (*Corvus corone*)‡	Crimea, Jan 2006 (*2*)
"Crows" (*Corvus* sp.)†	Japan, Mar 2004 (*1*)
**Rook** (*Corvus frugilegus*)‡	Crimea, Dec 2005 (*2*)
**Jackdaw** (*Corvus monedula*)‡	Crimea, Dec 2005 (*2*)
Common magpie (*Pica pica sericea*)‡	Hong Kong, Jan 2006 (*2*)
"Magpies" (Corvidae)†	Korea, Mar 2004 (*1*)
Oriental magpie robin (*Copsichus saularis*)‡	Hong Kong, Jan 2006 (*2*)
Red-billed mesia (*Leiothrix lutea*)†	Taiwan, Oct 2005 (*2*)
Crested myna (*Acridotheres cristatellus*)‡	Hong Kong, Jan 2006 (*2*)
"Mynas" (*Sturnidae*)**	Thailand, Oct 2005 (*2*)
Scaly-breasted munia (*Lonchura punctulata*)†	Thailand, Dec 2004 (*1*)
White-rumped munia (*Lonchura striata*)‡	Hong Kong, Jan 2006 (*2*)
Munia (*Lonchura* sp.)‡	Hong Kong, Jan 2006 (*2*)
Japanese white eye (*Zosterops japonica simplex*)‡	Hong Kong, Jan 2006 (*2*)
**Tree sparrow** (*Passer montanus*)†‡**	Hong Kong, Dec 2002; Thailand, 2005 (*1**–**3*)

*Those with known migratory populations are shown in **bold**. Underlined species had >1 seropositive test results in apparently healthy birds. Nomenclature follows the American Ornithologists' Union checklist (*7*) to the degree possible. Rasmussen and Anderton (*8*) is used as a supplementary source on this topic. †Old World species infected as captives or in association with captive birds in a zoological park, smuggler holding pen, or domestic poultry facility. ‡Old World species in which infection was detected at a site distant from any known infected domestic bird. §South American species, infected as a captive in a zoologic park. ¶North American species, infected as a captive in a zoologic park. #Australian species, infected as a captive in zoologic park. **Old World species; possible association with infected captive birds or poultry unknown.
